# Gas exchange, biomass and non-structural carbohydrates dynamics in vines under combined drought and biotic stress

**DOI:** 10.1186/s12870-019-2017-2

**Published:** 2019-09-18

**Authors:** Tadeja Savi, Almudena García González, Jose Carlos Herrera, Astrid Forneck

**Affiliations:** 1 Department of Integrative Biology and Biodiversity Research, University of Natural Resources and Life Sciences, Vienna (BOKU), Institute of Botany, Gregor-Mendel-Straße 33, 1190 Vienna, Austria; 2 Department of Crop Sciences, University of Natural Resources and Life Sciences, Vienna (BOKU), Institute of Viticulture and Pomology, Konrad Lorenz Strasse 24, A-3430 Tulln, Austria

**Keywords:** Abiotic stress, Water status, Herbivory, Phylloxera, *Vitis vinifera*, Riesling

## Abstract

**Background:**

Intensity of drought stress and pest attacks is forecasted to increase in the near future posing a serious threat to natural and agricultural ecosystems. Knowledge on potential effects of a combined abiotic-biotic stress on whole-plant physiology is lacking. We monitored the water status and carbon metabolism of a vine rootstock with or without scion subjected to water shortening and/or infestation with the sucking insect phylloxera (*Daktulosphaira vitifoliae* Fitch). We measured non-structural carbohydrates and biomass of different plant organs to assess the stress-induced responses at the root, stem, and leaf level. Effects of watering on root infestation were also addressed.

**Results:**

Higher root infestation was observed in drought-stressed plants compared to well-watered. The drought had a significant impact on most of the measured functional traits. Phylloxera further influenced vines water and carbon metabolism and enforced the sink strength of the roots by stimulating photosynthates translocation. The insect induced carbon depletion, reprogramed vine development, while preventing biomass compensation. A synergic effect of biotic-abiotic stress could be detected in several physiological and morphological traits.

**Conclusions:**

Our results indicate that events of water shortage favour insects’ feeding damage and increase the abundance of root nodosities. Root phylloxera infestation imposes a considerable stress to the plants which might exacerbate the negative effects of drought.

**Electronic supplementary material:**

The online version of this article (10.1186/s12870-019-2017-2) contains supplementary material, which is available to authorized users.

## Background

Climate change has increased the number of abiotic and biotic stressors in several ecosystems worldwide, impacting plant growth and production (Suzuki et al., 2014 [6];). Among abiotic stressors, variations in temperatures and rainfalls represent the biggest risk factor for survival of vegetation in both natural and agricultural ecosystems ([45]; Suzuki et al., 2014). Intense or prolonged drought usually leads to photosynthesis limitation, decreases the xylem water potential, and induces embolism formation with consequent reduction of water transport efficiency [22, 29, 43]. A large body of research on grapevines has improved our understanding of the physiological responses of different genotypes under drought [8, 25, 28]. However, beside the drought stress, insect pests and plant pathogens, in particular fungi and viruses represent a serious concern worldwide, since they exert an impact on vine physiology and health [6, 16, 33, 37]. The range of insect pests and pathogens can be influenced by climate change and their incidence is expected to further increase in the upcoming decades ([12, 38]; Suzuki et al., 2014 [6];) with consequent significant impacts on the winemaking sector (Suzuki et al., 2014 [16];).

Grape phylloxera (*Daktulosphaira vitifoliae* Fitch) is one of the most economically destructive and geographically widespread pests of commercial grapevines. It is a sucking and sedentary insect, obligate biotroph of *Vitis* species, native to North America where it coexists with native vine species [17, 24]. It was accidentally globally spread in the nineteenth century causing irreversible economic losses to the viticultural industry. Phylloxera-tolerant rootstocks with parentage of American *Vitis* species have been developed and successfully employed in the last century to buffer the detrimental effects of the pest [4, 37]. However, starting from the 80’s of the twentieth century, new outbreaks of phylloxera have been reported in Europe, Australia, South and North America endangering once again the viticulture [5, 37]. Moreover, rootstocks breakdown or failure due to the presence of more aggressive phylloxera biotypes has been also reported [15, 37].

Phylloxera feeds on parenchymal cells content and does not appear to penetrate vascular tissue [14, 24]. Different biotypes of the insect can attack root and/or leaf organs, but root-feeding stages are the most economically damaging [4, 15, 30, 37]. Feeding induces the formation of nodosities and tuberosities on young and mature roots, respectively ([17]; Battey and Simmonds, 2005). Root infestation apparently alters the water and nutrient absorbance capacity of plants [36], the pest competes for photosynthates leading to the enhancement of the carbon sink activity of roots [10, 14, 18, 23, 24]. Furthermore, wounding may produce entry points for soilborne pathogens which stimulate infections and secondary necrosis [23, 32] causing increased mortality of younger roots [3]. As a result of root damage, symptoms of intense phylloxera infestation are generally visible as reduced canopy vigour, premature leaf yellowing and smaller bunch size [4, 32]. Knowledge on how the different cultivars of vines respond to the root phylloxera attack, how the invasive insect influences whole-plant physiological functions, growth, and development is limited. Moreover, information on potential effects of a combined drought-root phylloxera stress on grapevine is particularly lacking in the scientific literature [2, 5]. In the light of prolonged drought periods coupled to high pests’ pressure, understanding and predicting the potential effects of a combined biotic-abiotic stress on vine represent a challenge for the future of viticulture.

To fill this knowledge gap, the present research project is aimed at investigating the physiology and carbon allocation of Riesling grafted on Teleki 5C, an economically important rootstock, under future “natural” growing conditions, characterized by prolonged drought periods and abundance of pests [6, 45]. Firstly, we wanted to shed light into the effects of watering on root phylloxeration. Secondly, we aimed to monitor the water status and whole-plant carbon metabolism of the vines subjected to water limitation or phylloxera infestation in order to improve our knowledge on the stress-induced plant responses at the root, stem, and leaf level. Furthermore, we wanted to assess, for the first time, eventual cumulative/synergic effects of the coupled biotic-abiotic stress on vine physiology, and highlight eventual compensation strategies of the host that might mitigate the damage and eventual negative effects of the pest on metabolism and development.

## Results

### Effects of the watering regime on root phylloxeration

After the acclimation period, the experimental vines were exposed to an eight-week long drought and/or phylloxera treatment. During this time interval the greenhouse temperatures averaged about 27 °C with minimum and maximum peaks of 10 °C and 46 °C, respectively. The average midday water pressure deficit oscillated between 0.6 and 5.5 kPa (Additional file 1: Figure S1). During the hottest hours of the day, the substrate temperatures were lower compared to those of the air by about 5 °C and 8 °C in D and W pots, respectively.

**Fig. 1:**
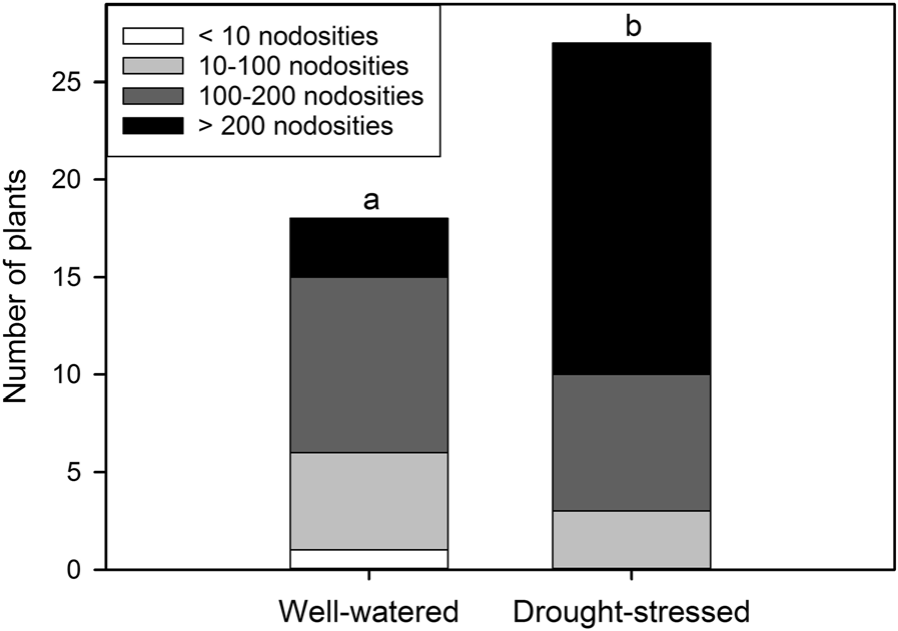
Root infestation evaluated at the end of the eight-week long treatment in well-watered and drought-stressed 5C and Riesling grafted on 5C grapevines (pooled data). Infestation intensity is classified as follows: 1 = presence of a low number of root nodosities (white stripes); 2 = 10 to 100 nodosities (light grey stripes); 3 = up to 200 nodosities (dark grey stripes); 4 > 200 nodosities (black stripes), presence of tuberosities on older lignified roots. Lettering denotes a statistically significant difference between groups (chi-square test)

The root infestation, analyzed at the end of the experiment, did not differ significantly between 5C and RR plants (same rootstock used); hence data were averaged and reported in Fig. 1. In well-watered vines (soil water content = 0.3 ± 0.04 g g^− 1^, W) the infestation frequency was 0.64, while in drought-stressed plants (soil water content = 0.1 ± 0.01 g g^− 1^, D) it increased up to 0.97. Compared to W vines, a significantly higher number of D plants showed intense infestation symptoms and fell in the assessment class “4” (> 200 nodosities, presence of tuberosities).

### Gas-exchange, water status and photosynthetic efficiency of the study vines

**Fig. 2:**
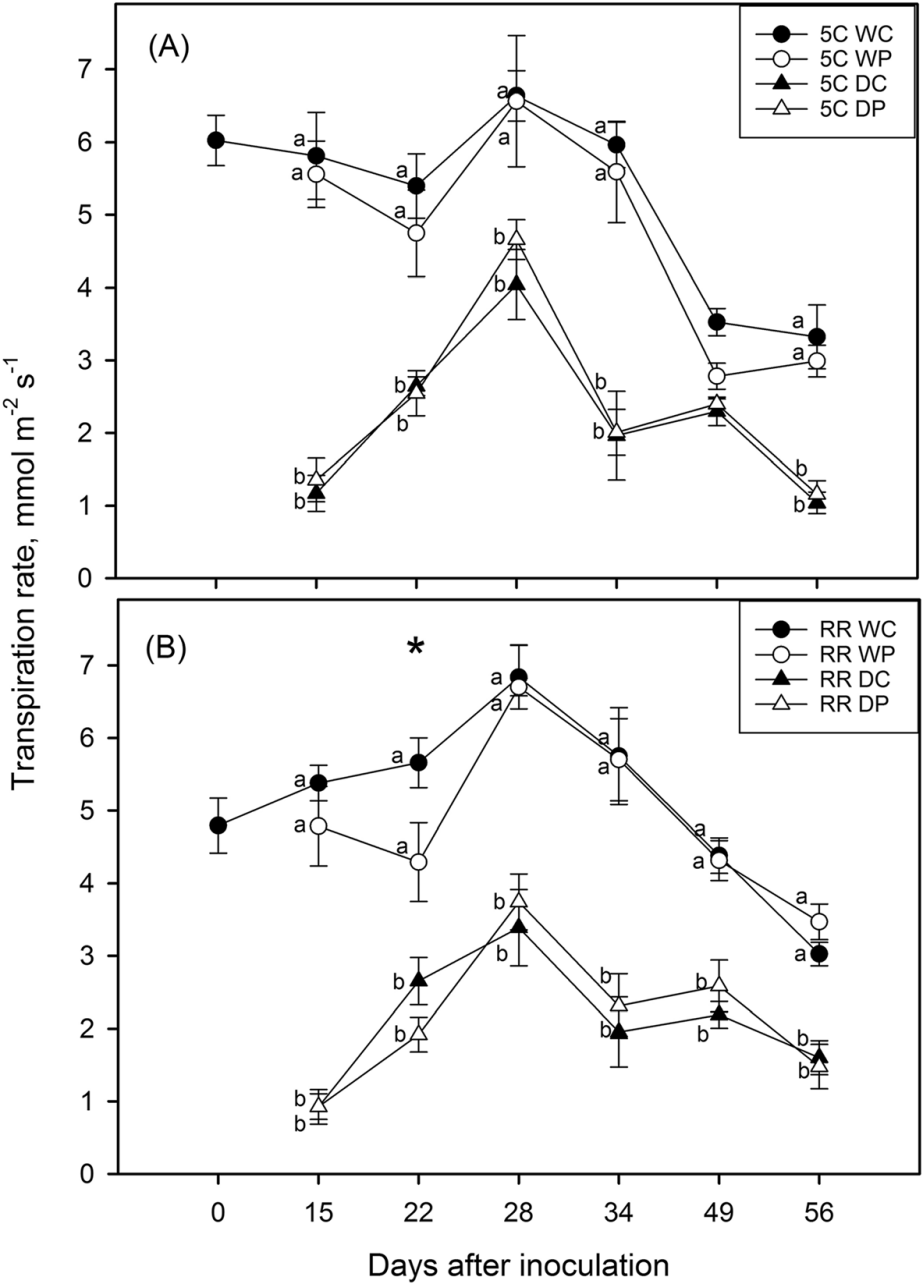
Transpiration rate (E_L_) measured in 5C (**a**) and Riesling grafted on 5C (**b**) during treatments application (*n* = 4–7). W = well-watered plants; D = drought-stressed; C = control, non-phylloxerated; P = root phylloxerated. Letters and asterisk indicate statistically significant difference within Irrigation (Factor I; W and D) and Infestation (Factor II; C and P), respectively. A significant interaction between factors (Irr x Inf) was observed in 5C on the 49th day after inoculation, i.e. within W level: C > P; within C level: W > D

**Fig. 3:**
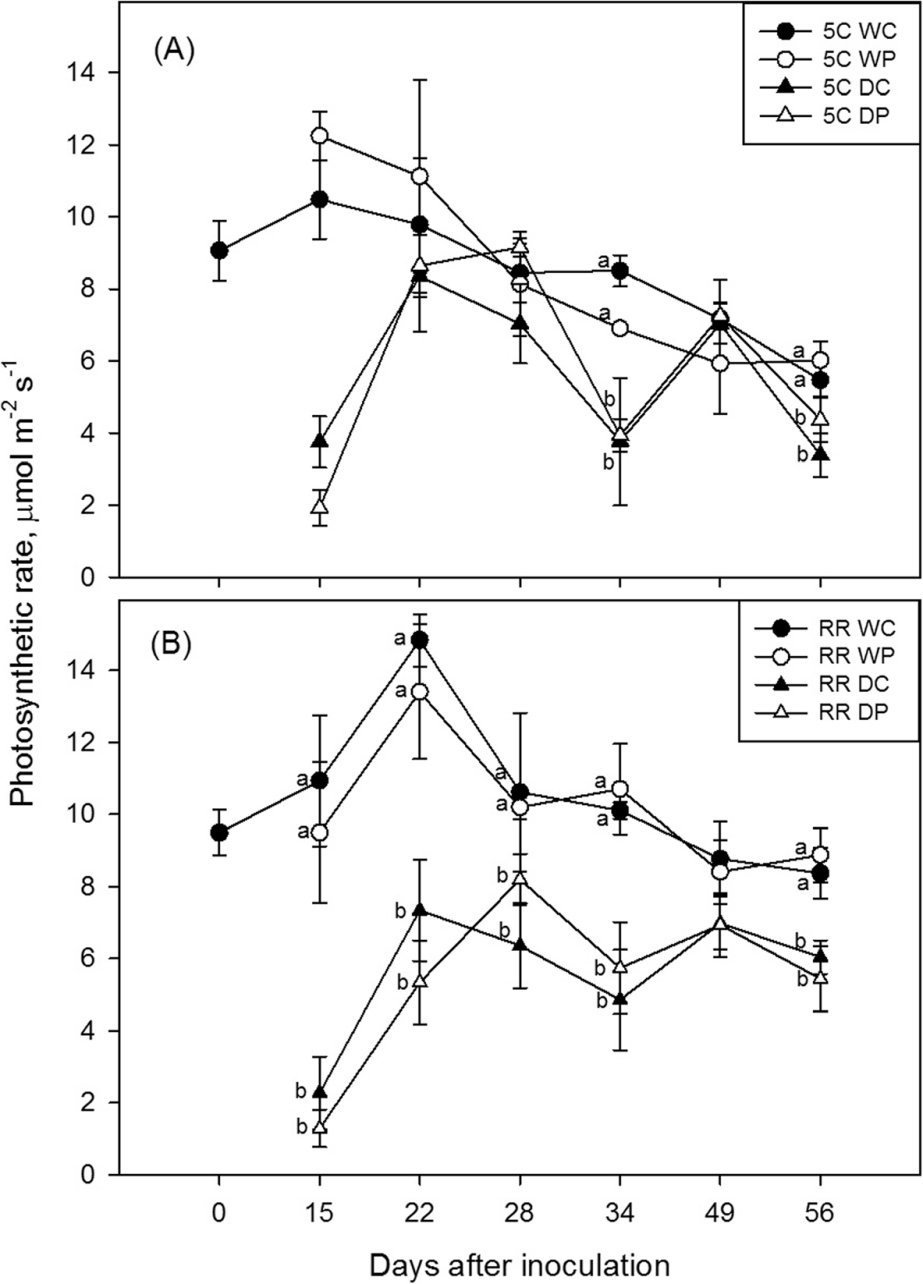
Photosynthetic rate (A) measured in 5C (**a**) and Riesling grafted on 5C (**b**) during treatments application (n = 4–7). W = well-watered plants; D = drought-stressed; C = control, non-phylloxerated; P = root phylloxerated. Letters denote statistically significant differences within Irrigation (Factor I; W and D), while n.s. indicates the lack of differences. A significant interaction between factors (Irr x Inf) was observed in 5C on 15th and 22nd day after stress imposition, i.e. within C level: W > D; within P level: W > D. No statistically significant differences within factor Infestation were observed

Figures 2 and 3 report the transpiration rate (E_L_) and net photosynthesis (A) measured in 5C (Figs. 2a and 3a) and Riesling grafted on 5C (Figs. 2b and 3b) plants on a weekly basis. The imposed moderate water deficit (ψ_stem_ and ψ_min_ of about − 1.0 and − 1.20 MPa, respectively; Table 1) significantly reduced both E_L_ and A. The stomatal conductance (g_s_) and sub-stomatal CO_2_ (ci) followed a similar trend to E_L_, while leaf temperature (T_leaf_) ranged between 32 °C and 40 °C, with the highest peaks recorded mostly in drought-stressed plants (Additional file 1: Figures. S2-S4). As expected, all traits were significantly affected by drought in both studied genotypes, even if photosynthetic rates and leaf temperature on a smaller extend compared to stomatal conductance, sub-stomatal CO_2_ and transpiration. On the other hand, phylloxera infestation did not induce notable shifts in the physiological traits, with the exception of E_L_ and g_s_ measured in RR vines three weeks after inoculation (30% lower values recorded in P than in C group), and ψ_min_ measured in 5C (Table 1) on the last sampling day (− 1.20 vs − 1.05 MPa measured in P and C group, respectively). Additionally, in 5C vines a significant interaction (Irrigation x Infestation) pointed out that in well-watered plants the E_L_ was, once again, 20% lower in P compared to C (Fig. 2a, 7 weeks after inoculation). The photosynthetic efficiency was apparently far less sensible to drought during the study period (data not shown), but a significant interaction between factors was highlighted in RR on the last sampling date (Table 1) with lower Fv/Fm displayed by the leaves under the biotic attack.

**Table 1:** Leaf temperature (T_leaf_), photosynthetic efficiency (Fv/Fm), stem and minimum water potential (ψ_stem_ and ψ_min_) measured in 5C and Riesling x 5C (RR) plants 8 weeks after the beginning of treatments (*n* = 5–8). W = well-watered plants; D = drought-stressed; C = control, non-phylloxerated; P = root phylloxerated. * indicates statistically significant differences within the factors (Irrigation or Infestation). A significant interaction between factors (Irr x Inf) was observed in Fv/Fm measured in RR, i.e. within W level: C > P; within P level: W < D

	5C					RR				
Traits	Irrigation		Infestation		Interaction	Irrigation		Infestation		Interaction
	W	D	C	P	Irr x Inf	W	D	C	P	Irr x Inf
Tleaf, °C	32.6 ± 0.5	33.5 ± 0.6	32.8 ± 0.5	33.3 ± 0.6	n.s.	**31.6 ± 0.6**	33.5 ± 0.7*	32.1 ± 0.6	33.0 ± 0.7	n.s.
Fv/Fm	0.80 ± 0.01	0.81 ± 0.01	0.80 ± 0.01	0.81 ± 0.01	n.s.	0.78 ± 0.01	0.79 ± 0.01	0.79 ± 0.01	0.77 ± 0.01	*
ψ_stem_, −MPa	**0.64 ± 0.03**	1.08 ± 0.03*	0.85 ± 0.03	0.87 ± 0.03	n.s.	**0.54 ± 0.04**	0.89 ± 0.04*	0.72 ± 0.04	0.71 ± 0.04	n.s.
ψ_min_, −MPa	**1.00 ± 0.04**	1.29 ± 0.04*	**1.08 ± 0.04**	1.20 ± 0.04*	n.s.	**0.82 ± 0.04**	1.10 ± 0.05*	0.97 ± 0.04	0.95 ± 0.05	n.s.

### Morphological traits of the study vines

Not surprisingly, the shoot length and aboveground biomass of both genotypes were higher in W plants than in D ones (Table 2). The phylloxeration in RR plants subjected to drought induced a slight reduction of both shoot length and biomass, but a significant difference was highlighted only in the roots’ dry biomass (19.9 ± 1.3 vs 23.8 ± 1.4 g for P and C, respectively; interaction Irrigation x Infestation). Moreover, the biotic threat reduced significantly the leaf mass allocation in RR plants (LMA of 5.6 ± 0.2 g cm^− 2^ in P and 6.1 ± 0.1 g cm^− 2^ in C group), but not in 5C.

**Table 2:** Shoot length, Leaf mass per area (LMA), dry biomass of leaf (DM_leaves_), stem (DM_stem_) and roots (DM_root_) measured for 5C and Riesling x 5C (RR) plants 8 weeks after the beginning of treatments (*n* = 7–10). W = well-watered plants; D = drought-stressed; C = control, non-phylloxerated; P = root phylloxerated. * indicates statistically significant differences within the factors (Irrigation or Infestation). A significant interaction between factors (Irr x Inf) was observed in Shoot length (within C level: W > D; within P level: W > D) and DM_roots_ (within D level: C > P) measured in RR

	5C					RR				
Traits	Irrigation		Infestation		Interaction	Irrigation		Infestation		Interaction
	W	D	C	P	Irr x Inf	W	D	C	P	Irr x Inf
Shoot length, m	**5.3 ± 0.2**	2.0 ± 0.2*	3.8 ± 0.2	3.5 ± 0.2	n.s.	**3.5 ± 0.1**	1.4 ± 0.1*	2.4 ± 0.1	2.5 ± 0.1	*
LMA, g cm^−2^	5.7 ± 0.1	5.6 ± 0.1	5.6 ± 0.1	5.7 ± 0.1	n.s.	5.8 ± 0.1	5.9 ± 0.2	**6.1 ± 0.1**	5.6 ± 0.2*	n.s.
DM_leaves_, g	**31.0 ± 1.6**	13.9 ± 1.5*	23.0 ± 1.7	21.9 ± 1.4	n.s.	**23.7 ± 0.9**	11.4 ± 0.9*	17.6 ± 1.0	17.4 ± 0.9	n.s.
DM_stem_, g	**26.8 ± 1.3**	9.1 ± 1.2*	18.7 ± 1.3	17.2 ± 1.2	n.s.	**22.5 ± 1.0**	9.0 ± 0.9*	15.7 ± 1.0	15.8 ± 0.9	n.s.
DM_roots_, g	21.2 ± 1.3	23.8 ± 1.2	22.0 ± 1.3	23.1 ± 1.2	n.s.	22.2 ± 1.0	21.9 ± 1.0	22.6 ± 1.0	21.5 ± 1.0	*

### Non-structural carbohydrates (NSC) content

NSC measured in leaves, stems, and roots are summarized in Table 3 (a-c). Overall, the concentration of soluble carbohydrates was relatively higher in roots, followed by leaves and stems, while starch was much more abundant in roots and stems than in leaves. All plants subjected to drought showed a lower glucose and fructose concentration in the leaves (by about 40% compared to well-watered), but the starch content was negatively affected only in RR. At the stem level, the water scarcity led to a decrease of glucose and fructose in 5C plants, while an opposite trend was observed in RR. Significant differences in sucrose (the major transport sugar form) were observed only at the root level, where it peaked up to 95 mg g^− 1^ in the D group and reached only 78 mg g^− 1^ in W. Once again, the drought led to a depletion of glucose and starch in the roots of RR plants, but not of 5C.

**Table 3:** Glucose, fructose, sucrose, and starch concentrations measured in leaves (a), stems (b) and roots (c) of 5C and Riesling x 5C (RR) 8 weeks after the beginning of treatments (n = 5–8). W = well-watered plants; D = drought-stressed; C = control, non-phylloxerated; P = root phylloxerated. * indicates statistically significant differences within the factors (Irrigation or Infestation). At root level, a significant interaction between factors (Irr x Inf) was observed in sucrose measured in 5C (within D level: C > P; within C level: W < D) and glucose (within W level: C > P; within C level: W > D) measured in RR

	5C					RR				
	Irrigation		Infestation		Interaction	Irrigation		Infestation		Interaction
**(a)** Leaves	W	D	C	P	Irr x Inf	W	D	C	P	Irr x Inf
Glucose, mg g^− 1^	**24.2 ± 2.7**	15.1 ± 2.8*	17.7 ± 2.8	21.7 ± 2.7	n.s.	**14.7 ± 1.1**	9.2 ± 1.1*	12.3 ± 1.1	11.6 ± 1.1	n.s.
Fructose, mg g^− 1^	**31.6 ± 3.8**	18.7 ± 3.8*	21.5 ± 3.9	28.8 ± 3.7	n.s.	**13.7 ± 1.1**	8.6 ± 1.2*	11.3 ± 1.1	11.0 ± 1.2	n.s.
Sucrose, mg g^− 1^	101.5 ± 12.7	99.5 ± 11.9	89.7 ± 13.0	111.4 ± 11.5	n.s.	111.2 ± 9.6	110.4 ± 10.0	115.1 ± 10.0	106.4 ± 9.6	n.s.
Starch, mg g^− 1^	2.5 ± 0.4	3.4 ± 0.4	3.1 ± 0.4	2.7 ± 0.4	n.s.	**9.1 ± 1.0**	3.9 ± 1.1*	**8.2 ± 1.0**	4.8 ± 1.1*	n.s.
**(b)** Stems	W	D	C	P	Irr x Inf	W	D	C	P	Irr x Inf
Glucose, mg g^− 1^	**7.6 ± 0.6**	4.1 ± 0.6*	5.7 ± 0.6	6.1 ± 0.6	n.s.	**3.3 ± 0.4**	4.9 ± 0.4*	4.4 ± 0.4	3.8 ± 0.4	n.s.
Fructose, mg g^− 1^	**4.7 ± 0.3**	2.5 ± 0.3*	3.7 ± 0.3	3.5 ± 0.3	n.s.	**1.7 ± 0.1**	2.1 ± 0.1*	1.9 ± 0.1	1.9 ± 0.1	n.s.
Sucrose, mg g^− 1^	95.7 ± 4.6	93.4 ± 4.6	96.0 ± 4.8	93.1 ± 4.5	n.s.	80.4 ± 4.5	87.2 ± 4.5	84.0 ± 4.5	83.6 ± 4.5	n.s.
Starch, mg g^− 1^	11.5 ± 1.7	15.9 ± 1.8	15.1 ± 1.8	12.3 ± 1.7	n.s.	**8.4 ± 1.4**	19.1 ± 1.4*	13.8 ± 1.4	13.7 ± 1.4	n.s.
**(c)** Roots	W	D	C	P	Irr x Inf	W	D	C	P	Irr x Inf
Glucose, mg g^−1^	74.3 ± 8.3	60.1 ± 8.0	**101.2 ± 8.0**	33.1 ± 8.3*	n.s.	**72.8 ± 7.4**	38.0 ± 8.1*	**84.1 ± 8.1**	26.8 ± 7.4*	*
Fructose, mg g^− 1^	17.5 ± 2.0	19.5 ± 1.9	**27.6 ± 1.9**	9.4 ± 2.0*	n.s.	13.4 ± 1.8	13.7 ± 1.9	**19.2 ± 1.9**	7.9 ± 1.8*	n.s.
Sucrose, mg g^− 1^	**77.7 ± 6.2**	95.3 ± 6.0*	94.5 ± 6.0	78.5 ± 6.2	*	72.7 ± 5.0	93.7 ± 5.0*	**98.7 ± 5.2**	67.6 ± 4.8*	n.s.
Starch, mg g^−1^	18.7 ± 2.9	17.1 ± 3.0	15.3 ± 2.9	20.4 ± 3.0	n.s.	**21.7 ± 2.7**	13.2 ± 2.9*	18.3 ± 2.9	16.6 ± 2.7	n.s.

Apparently, phylloxeration didn’t induce marked shifts of NSC in the aboveground organs of both genotypes studied (Table 3 a, b), with the exception of a sharp reduction of starch in RR leaves (4.8 ± 1.1 and 8.2 ± 1.0 mg g^− 1^, measured in P and C plants respectively). On the other hand, roots responded to the biotic attack with a significant reduction (by about 50%) of all soluble sugars (Table 3c). Furthermore, a significant interaction between irrigation and infestation was highlighted for the root glucose measured in RR and sucrose measured in 5C.

## Discussion

Under climate change, the spread and accelerated reproduction of phylloxera represent a potential threat for the wine industry. We monitored the whole-plant response to abiotic and/or biotic stress through measurements of vine water and carbon metabolism, carbohydrate partitioning, and below/aboveground biomass allocation. We found evidence that the combination of drought and herbivory stress significantly impact water status and carbon allocation of grapevines.

Teleki 5C turned out to be a tolerant rootstock able to support the proliferation of phylloxera on nodosities. Despite the high temperatures recorded during the study period with daily maximum above 45 °C (Additional file 1: Fig. S1, substrate temperatures above 40 °C), the root inoculation was successful in 84% of plants, suggesting that previously reported optimal ambient temperatures for phylloxera survival and gall formation ranging between 22 and 30 °C [17, 37] should be re-considered and/or potentially heat-resistant phylloxera biotypes have evolved. It has been suggested that, apart from the temperature, a range of other abiotic factors including type of the soil, seasonality and humidity [7, 17, 37] influence the survival and reproduction of phylloxera and its consequent impacts on host plants. With regard to soil moisture, the phylloxera population in our trial did not develop in 3 and 36% of drought-stressed and well-watered plants, respectively. It is worth noting that well-watered pots had a water content of about 0.3 g g^− 1^, hence far lower than the soil saturated water content (0.51 g g^− 1^), indicating that the roots were not subjected to waterlogging which might reduce the insect proliferation [37]. The root system of drought-stressed vines was significantly more infested showing numerous nodosities compared to W ones (Fig. 1). We want to point out that the maximum substrate temperatures were by about 3 °C higher (differences not statistically significant) in D pots compared to W ones (data not shown). Our data, hence, suggests that the water scarcity may exacerbate the biotic stress, by increasing the insect’s feeding damage/number of nodosities on plant roots. Interestingly, this result is not in accordance with the only other study found in the literature addressing the effects of watering on phylloxeration of vines [2] in which a greater gall density was reported for irrigated compared to non-irrigated plants in the pots. The contrasting plant-insect response to water scarcity observed in the two studies might be driven by several factors differing in the two experimental set-ups, i.e. microclimate, substrate characteristics (porosity, oxygen diffusion), competing soil fauna and flora, root architecture, drought/phylloxera tolerance of the used rootstocks and insect biotypes etc. [3, 12, 15].

As expected, the drought stress had a significant negative impact on most of the measured functional traits. Both genotypes displayed a prompt stomatal closure, but Riesling scion buffered more efficiently the drop in the water potential (isohydric water strategy, [31]), showing at the same time a lower photosynthetic efficiency compared to 5C (Table 1). At first sight, phylloxeration did not cause shifts in the leaf functionality throughout the study period. However, a general trend toward lower transpiration rates and stomatal conductance values in the P group compared to C ones could be noticed in both genotypes, and especially in well-watered plants. The differences were found to be statistically significant on two out of six dates of measurements (Fig. 2 and Additional file 1: Fig. S2) indicating that root infestation may impact, even if on a small extend, the water status and gas exchange of the aboveground organs. Under drought conditions, all vines were water-stressed and the effect of the pest could be detected earliest three weeks after inoculation and only in RR plants. As a likely consequence of high data variability and the lower sensibility of net photosynthesis to stress factors [21, 28], differences in A between the infestation levels were not evident. On the other hand, a significant drop of the maximum quantum yield of PSII after dark adaptation could be observed in well-watered Riesling plants at the peak of the treatments (Fv/Fm of 0.81 vs 0.74 measured in C and P, respectively), suggesting the occurrence of insect-induced perturbations in the photosynthetic apparatus [13, 42].

The leaf mass per area ratio (LMA) is often used to study the biomass allocation and productivity gradients within the aboveground organs under different stressors, since it scales positively with the carbon investments in secondary compounds such as tannins and lignins (Pérez-Harguindeguy et al., 2016 [35, 42];). Phylloxera infestation apparently increased the stress experienced at the leaf-level leading to a reduction of LMA in the Riesling scion. This would suggest that, despite similar photosynthetic rates, phylloxerated RR undergoes less carbon investment per unit leaf area construction compared to control. Similarly to LMA, the biotic attack coupled to drought stress significantly reduced the root biomass of RR and limited the shoot length by about 20% (1.2 vs 1.5 m as measured in DP and DC plants, respectively). Hence, compensatory growth in response to root herbivory suggested by other authors [10, 23] was not highlighted in our study at the aboveground, neither at the belowground level. The lower primary metabolites allocation in biomass observed in Riesling might be a direct consequence of i) the carbon export toward the phylloxera population which has been suggested that acts as a strong sink [24, 46], as well as ii) an effect of the redirection of energy in the secondary metabolic pathways (defense, repair, signaling, phytohormonal networks [11, 23];). In fact, it has been recently demonstrated that plants tend to accelerate carbon allocation for defense under stress synthetizing a large amounts of secondary metabolites, i.e. organic volatiles, flavonoids, stilbenes etc. [1, 11, 27], a process that requires conspicuous energy investment [23, 47]. Apparently, 5C plants showed a higher tolerance to the biotic attack compared to the grafted Riesling, although a slight and not significant reduction of shoot length and aboveground biomass could be observed also in these plants.

To our knowledge, the present study addresses for the first time, the whole-plant NSC dynamics under phylloxera and drought stress. The values of NSC measured in leaves, stems, and roots are in overall agreement with data reported in the literature for grapevine [20, 39, 44]. The drought treatment led to a general depletion of monosaccharides in both above and below-ground organs, with the exception of RR stems where glucose and fructose increased as a likely consequence of late summer sugars translocation toward perennial organs characterizing this less vigorous cultivar. However, the high sucrose concentration observed in all plants and organs indicates that phloem activity and translocation was still intense. The depletion of NSC due to drought stress has been already reported and linked to stomatal closure and reduced carbon fixation coupled to the increased energetic demand for respiration, basal metabolism, osmoregulation, and synthesis of defense compounds [19]. In our study, the water limitation caused a 40% starch reduction (main storage compound) in roots of grafted Riesling, but not in own-rooted 5C. On the basis of the above we can speculate that the more isohydric behavior of RR plants induced an imbalance between carbon uptake via photosynthesis and carbon substrate demand (respiration, turgor maintenance, defense, repair) leading to a massive loss of carbohydrates [19, 29] and consequent reduction of biomass.

Apparently, infestation did not influence NSC levels in the aboveground organs, with the exception of a marked reduction of starch found in the leaves of Riesling. Similarly to what observed in morphological traits, this result supports the idea that the insect actively limits carbon allocation and storage in the leaves, while likely stimulates its translocation toward galling habit or/and its usage in alternative metabolic pathways. Interestingly, differences in starch concentration were not observed in the stem tissue (about 13.8 mg g^− 1^ in both C and P groups). However, at the root level, significant reductions of all soluble sugars were recorded when comparing phylloxerated and control plants. These results might suggest that in phylloxerated plants the sugars are redirected elsewhere, as for example in the synthesis of defense compounds in response to herbivory. Furthermore, we can also speculate that the carbon reserves are, as least partially, withdrawn by the insect population [10, 18, 46]. The synergic effect of phylloxera and water stress was highlighted for sucrose and glucose measured in 5C and RR respectively; indicating that the infestation may interfere with water uptake exacerbating the water stress. The absence of statistically significant differences in starch between the infestation levels may be due to the harsh environmental conditions of the greenhouse (high temperatures and water pressure deficits) which limited carbon fixation and storage in all study plants, as well as a consequence of high data variability and relatively limited number of replicates (*n* = 5–8). Similarly, Ryan et al. [39] failed in pointing out clear differences in starch concentration between infested and uninfested roots, while other authors reported a general increase of it [18, 24]. However, a closer look to our data reveals that the infested root-tips of 5C plants contained higher (but not significant) concentration of starch compared to C, especially when subjected to drought stress. This might indicate that Teleki 5C genotype better tolerates root infestation compared to RR by maintaining certain carbon storage in the roots.

To better address the effect of phylloxeration on the whole-plant carbon metabolism, we estimated the theoretical total amount of carbohydrates available in the experimental vines by multiplying the NSC with the dry mass of different organs. This rough calculation showed that control roots in both genotypes contained about 5 g of NSC, while in infested roots the value was limited to only 3 g. Interestingly, at the aboveground level RR plants showed a depletion of total available NSC (− 6%), while 5C plants had similar values in C and P groups. However, when the whole plant organism (leaves, stems, roots) was considered, the carbon availability of the phylloxerated group was by 30% (RR) and 15% (5C) lower compared to control ones. These results confirmed, once again, that the pest influences the mobility of metabolites between above and below ground organs leading to a general reduction of carbon resources. Extending the research on secondary metabolites produced by plants under different stresses would shed light on the fraction of nutrients that is redirected to sustain plants defense mechanisms and that which is actually withdrawn by the insect population.

## Conclusion

Our experiment represents the most complete report on the whole-vine response to root infestation of phylloxera. Through measurements of leaf physiology, we demonstrated that the insect is able to impact water and carbon metabolism of plants, likely enforcing the sink strength of the roots by stimulating the carbon translocation in favor of the galling habit and/or redirecting the sugars toward defense metabolic pathways. Moreover, we showed that phylloxera reprograms vine carbon allocation and development, while preventing or limiting biomass compensation. Furthermore, to our knowledge this is the first study addressing the combined effects of drought and phylloxera on vine physiology. A more intense root infestation was observed in drought-stressed plants compared to watered ones, suggesting that events of water shortage favor the insect’s feeding damage. The synergic effects of biotic-abiotic stress on vine could be clearly detected in physiological traits, as well as in shoot length and root biomass. In particular, significant differences in LMA, roots’ dry mass, and sugars concentration suggested that belowground infestation imposes a considerable stress to the plants, which might exacerbate the negative effects of drought. Teleki 5C is a tolerant rootstock supporting large population of phylloxera, but efficiently buffering negative effects on plant development. The overall more marked response of grafted Riesling compared to own-rooted 5C, indicates a higher sensitivity to phylloxera of the former genotype, and demonstrates the potential ability of the scion in influencing the whole-plant physiology.

## Methods

### Plant material and experimental set-up

The study was carried out in the greenhouse of the Institute of Viticulture and Pomology, BOKU, Tulln (Lower Austria) in spring-summer 2018. As host plant material the rootstock Teleki 5C (*V. riparia* x *V. berlandieri*) without or with grafted Riesling scion (5C and RR, respectively) were selected. Plants were provided by a nursery (Reben IBY KEG) where the formal identification of the plant material was undertaken. A voucher specimen of this material has not been deposited in a publicly available herbarium. At the beginning of April, 50 plants of 5C (one year old) and 50 of RR (two years old) were potted in seven liters pots containing a mixture of natural soil (chernozem) collected from a nearby site ([9] Digitale Bodenkarte Österreich; https://gis.bmnt.gv.at/eBOD) and perlite (Premium Perlite, Gramoflor; 70:30). The water content at field capacity of the mixture, defined as the amount of water content held in the soil after excess water has drained away, was 0.51 ± 0.03 g g^− 1^. The plants were fertilized (ENTEC vino, EuroChem Agro GmbH; 23 g per pot) and maintained in well-watered conditions by drip irrigation. After about eight weeks of growth, the experimental plants were trimmed to uniform the canopy size by leaving 15 leaves (total shoot length of about 75 cm). One week after, half of the plants per genotype were root-inoculated with 100 phylloxera eggs (P) collected from a field population developing on leaves of adult 5C plants. The non-infested plants were considered as control (C). All the pots were enclosed and accurately sealed in a bag made of polypropylene tissue (mesh 125 μm) preventing the spread of phylloxera.

The day after inoculation, the pots were randomly disposed equally spaced, on tables and subdivided into two additional groups per infestation category, i.e. well-watered plants (W) and drought-stressed (D) plants. W vines were irrigated daily with about 150 ml of water. The drought treatment consisted of the progressive reduction of irrigation volumes aimed at reducing the leaf stomatal conductance at values corresponding to about 30% of those recorded in W plants (moderate water deficit). Soil water content was measured at the end of the treatment. About 8 h after the last irrigation, 5 plants per experimental category (W and D) were randomly selected, 5–8 g of soil was collected from the central part of the pots and the fresh weight measured. The samples were then placed in an oven (48 h at 45°) to get their dry weight. The soil water content was calculated as (fresh weight - dry weight) / dry weight, and expressed in g g^− 1^.

To summarize, the experimental set up consisted on a full 2 × 2 factorial design with Irrigation and Infestation as main factors and their combinations, for a total of four treatments per genotype, i.e. well-watered not infested (WC), well-watered phylloxerated (WP), drought-stressed not infested (DC) and drought-stressed phylloxerated plants (DP). The treatments were maintained for eight weeks.

Air temperature and relative humidity were recorded on an hourly basis during the whole study period using three data-loggers (UT330B, Uni-trend Technology, Hong Kong) installed at 1.5 m and facing North. To evaluate the atmospheric evaporative demand, average midday water pressure deficit (kPa) was calculated as the difference between the actual amount of moisture in the air and the maximum moisture that the air hold at saturation. Substrate temperatures were measured with mini data-loggers (Thermochron iButton, iButtonLink, LLC, Whitewater, WI) installed at a depth of about 20 cm of three experimental pots per watering category (six pots in total).

### Physiological measurements and sampling

In order to adjust the watering, maintain a moderate degree of drought stress in D plants, and assess eventual differences among experimental treatments, stomatal conductance to water vapor (g_s_), transpiration rates (E_L_), net photosynthesis (A), sub-stomatal CO_2_ (ci), leaf surface temperature (T_leaf_), and photosynthetic efficiency (Fv/Fm) were measured weekly during the eight-week long treatment. Measurements were performed between 11 and 14 h on at least one mature fully expanded and undamaged leaf per plant. At least five randomly selected plants per genotype and experimental treatment were measured in each day, for a total of six dates. g_s_, E_L_, T_leaf_, and A were measured with the gas-exchange system LCpro-SD (ADC BioScientific Ltd., Hertfordshire, UK). The Fv/Fm was recorded on dark-adapted leaves as a quantitative measure of the maximum efficiency of PSII using a portable fluorimeter (Handy Pea, Hansatech, Norfolk, UK). The parameter has been widely used as a sensitive indicator of photosynthetic performance under stress conditions [13, 40].

During measurements, the air temperature and relative humidity in the greenhouse averaged about 32 °C and 40%, respectively, while the photosynthetic photon flux density ranged between 600 and 1000 μmol m^− 2^ s^− 1^. For details on climatic parameters see Additional file 1: Figure S1.

Eight weeks after the beginning of treatments, at the peak of the abiotic and biotic stress, physiological measurements were performed on six to eight plants per experimental category. In addition, minimum (ψ_min_) and stem water potentials (ψ_stem_) were assessed by means of a Scholander pressure chamber (3000 Series Plant Water Status Consoles, Soilmoisure, Santa Barbara, CA [41];) sampling the closest leaves to the one used for photosynthesis measurements. The leaf for ψ_min_ was collected, wrapped in cling film, inserted in a sealed plastic envelope, and stored in a cool bag. On the other hand, ψ_stem_ was assessed in leaves that were bagged in cling film and covered with aluminum foil two hours before sampling. All leaves were measured within two hours after sampling.

Furthermore, right after noninvasive gas-exchange measurements, the leaves were sampled, snap frozen in liquid nitrogen and stored at − 80 °C for subsequent non-structural carbohydrate (NSC) analyses (see below). A 5 cm long stem segment cut from the central part of the shoot and a root sample of about 2 g (15 mm long root tips) were also collected from each plant and stored as described above. The roots were previously abundantly rinsed with distilled water to remove soil particles and insects.

### Assessment of phylloxera infestation

With the aim to highlight eventual differences in the root infestation of P vines among the experimental treatments, the plants were gently uprooted and the roots observed under a stereo microscope. Two integrated parameters were calculated [36]. In particular, the infestation frequency was defined as the ratio of inoculated plants showing infestation symptoms and the total number of actually inoculated vines. The infestation intensity was further classified into four assessment classes, i.e. 1 = presence of a low number of root nodosities; 2 = 10 to 100 young (white to light-yellow color) nodosities; 3 = up to 200 young and old (dark brown) nodosities; 4 > 200 nodosities, developed on older lignified roots, as well [36]. P plants that did not show signs of insect infestation were excluded from all the experimental measurements and sampling.

### Measurements of morphological traits

In order to study the biomass allocation in the experimental vines, additional morphological measurements [34] were performed on the plant material (*n* = 7–10). The two leaves used for ψ measurements (see above), were re-hydrated overnight by immersing their petiole in distilled water while wrapped in cling film. The petiole was then cut, the leaf blades scanned, and the area measured with imageJ (https://imagej.nih.gov/ij). The mass of each leaf was recorded after drying (48 h at 70 °C) and the LMA (leaf mass per area) calculated as dry mass / leaf area (Pérez-Harguindeguy et al., 2016).

The stem of all plants was cut at the root collar. The root systems were gently washed with tap water in order to remove residual soil particles and oven-dried (72 h at 70 °C) to get the dry biomass (DM_roots_). The absolute shoot length was measured with a meter tape, the leaves were detached and the dry mass of the two organs (DM_stems_ and DM_leaves_) was recorded after drying.

### Non-structural carbohydrates (NSC) analyses

To verify whether the stressors influence carbon allocation/translocation dynamics in different experimental categories, NSC (major source of energy for plants, [47]) were measured at the end of the treatments. After being ground to fine powder in liquid nitrogen and freeze-dried for 48 h, about 50 mg of the leaf, root, and stem samples were used to measure the NSC concentration according to Landhäusser et al. [26]. Total soluble sugars were extracted in 80% ethanol (three boiling cycles) and the supernatant filtered at 0.45 μm nylon syringe filter. The collected supernatant from the three extractions was pooled together. After necessary dilution, the samples were analyzed with anion exchange chromatography (Dionex™ ICS-5000, Thermo Fischer Scientific, Waltham, MA) using a Dionex CarboPac™ PA20 column (3 × 150 mm) coupled with a Dionex CarboPac™ PA20 guard column (3 × 30 mm) kept at 30 °C and using NaOH 52 mM as eluent under isocratic conditions (flow rate 0.5 ml/min). Glucose, fructose and sucrose were quantified using their reference standards with the software Chromeleon (v 7.2, Thermo Scientific, Waltham, MA). The starch contained in the pellet was converted to glucose with α-amilase (70 units per sample, 1 ml) followed by amyloglucosidase (6 units per 0.1 ml of sub-sample; for details see [26]). After digestion of the pellet, the samples were treated with chloroform (1:1 v/v), the aqueous layer was filtered, and the glucose hydrolysate measured as described above.

### Data analyses

Statistical analysis was performed in R (v 3.5.1) and SigmaPlot (v 13, Systat Software Inc., San Jose, CA). The effect of different treatments was tested separately for each parameter and for each genotype independently. Normality of data distribution was tested using the ‘qqPlot’ function (‘CAR’ package). A General Liner Model was run to detect differences among values of traits measured in the four experimental categories per genotype. Each trait was considered as a response variable, while Infestation and Irrigation were treated as explanatory variables (factors). To detect statistically significant differences among groups a multiple comparison procedure based on the Holm-Sidak method was run on the data. The categorical root-infestation data recorded for P plants was analyzed using chi-square test. All results were considered statistically significant at *P* ≤ 0.05. Mean ± standard errors of the mean are reported.

## Supplementary information

Additional file 1: **FigureS1.** Microclimatic data recorded in the greenhouse during the experimental period: average midday water pressure deficit (grey area, right axis), minimum (closed circles) and maximum (open circles) daily temperatures (left axis). The two arrows indicate the inoculation and the final sampling days, respectively. **Figure S2.** Stomatal conductance to water vapor (g_s_) measured in 5C (a) and Riesling grafted on 5C (b) during treatments application (*n* = 4–7). W = well-watered plants; D = drought-stressed; C = control, non-phylloxerated; P = root phylloxerated. Letters and asterisk indicate statistically significant difference within Irrigation (Factor I; W and D) and Infestation (Factor II; C and P), respectively. No statistically significant interaction between factors was observed. **Figure S3.** Leaf temperature (T_leaf_) measured in 5C (a) and Riesling grafted on 5C (b) during treatments application (n = 4–7). W = well-watered plants; D = drought-stressed; C = control, non-phylloxerated; P = root phylloxerated. Letters denote statistically significant differences within Irrigation (Factor I; W and D). No statistically significant differences within factor Infestation or interaction between factors were observed. **Figure S4.** Sub-stomatal CO_2_ (Ci) measured in 5C (a) and Riesling grafted on 5C (b) during treatments application (n = 4–7). W = well-watered plants; D = drought-stressed; C = control, non-phylloxerated; P = root phylloxerated. Letters denote statistically significant differences within Irrigation (Factor I; W and D). No statistically significant differences within factor Infestation or interaction between factors were observed.

## Data Availability

The datasets used and analyzed during the current study are available from the corresponding author on reasonable request.

## Abbreviations

A: net photosynthesis
ci: sub-stomatal CO_2_
DM: dry biomass
E_L_: transpiration rates
Fv/Fm: photosynthetic efficiency
g_s_: stomatal conductance to water vapor
LMA: leaf mass per area
NSC: non-structural carbohydrates
T_leaf_: leaf surface temperature
ψ_min_: minimum water potential
ψ_stem_: stem water potential
