# Supplementary figures and images for: Gas exchange, biomass and non-structural carbohydrates dynamics in vines under combined drought and biotic stress
Source: BMC Plant Biol. 2019 Sep 18;19:408. doi: 10.1186/s12870-019-2017-2 (PMC6749654; doi:10.1186/s12870-019-2017-2)

SUPPORTING INFORMATION


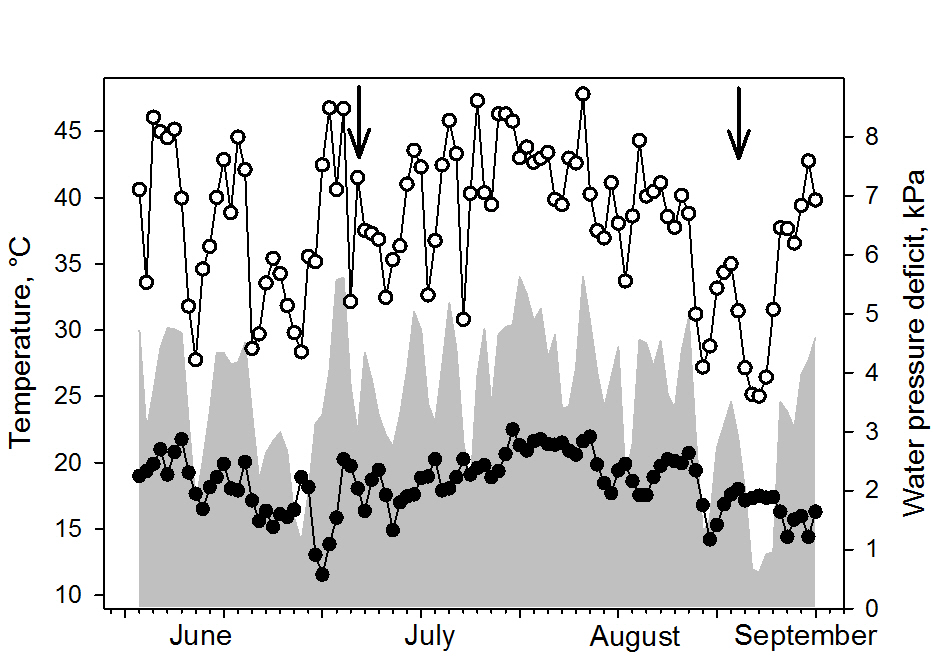


Figure S1


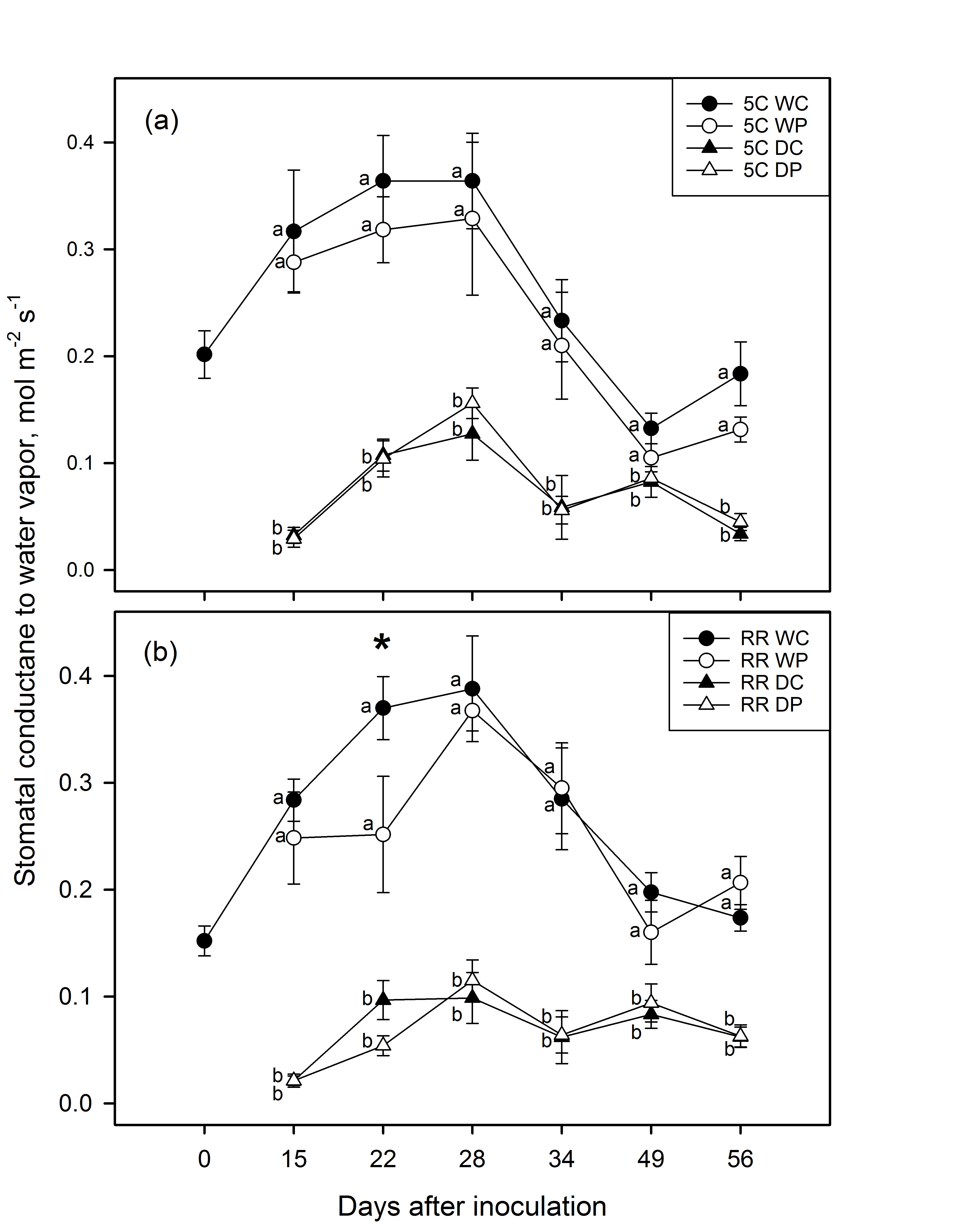


Figure S2


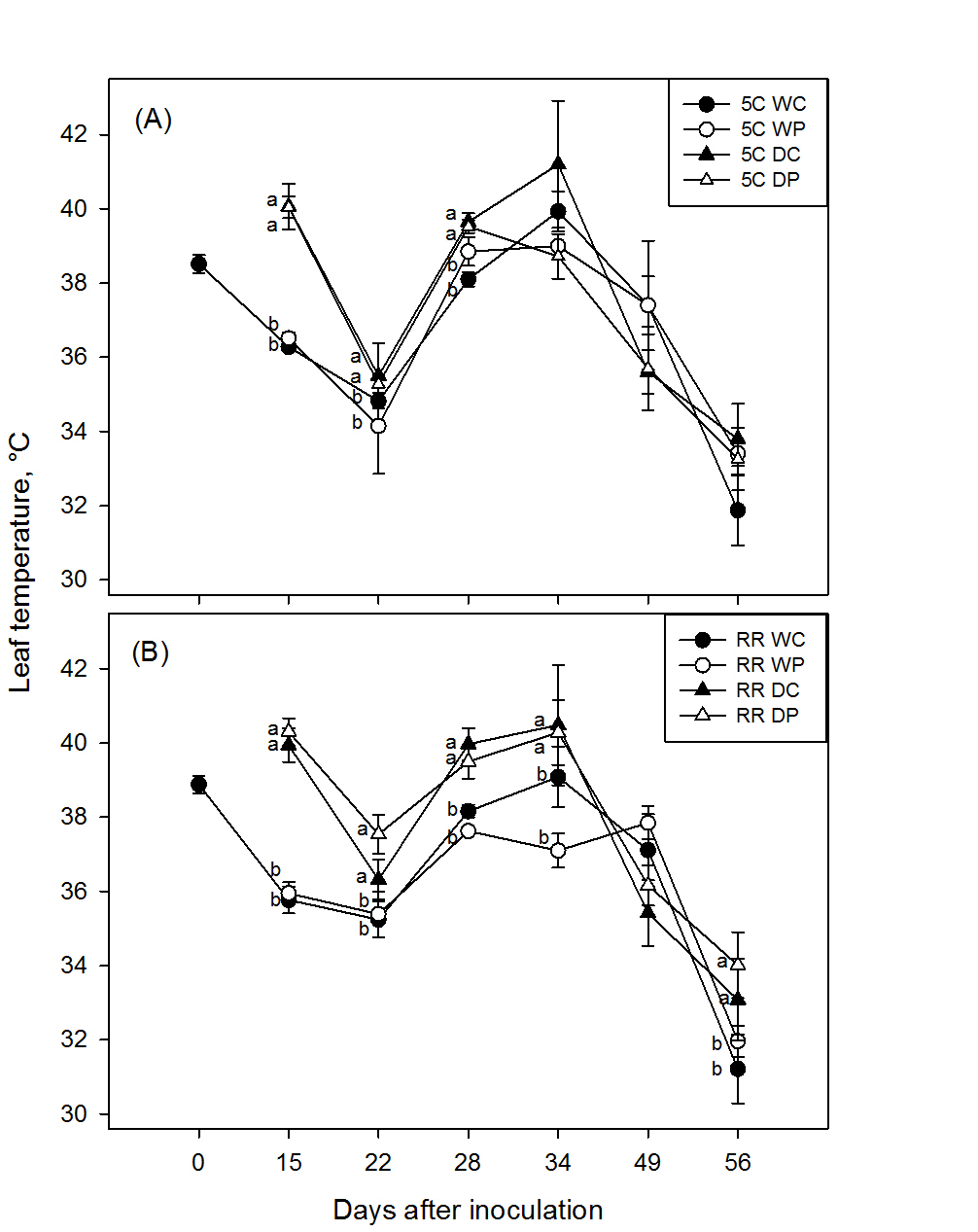


Figure S3


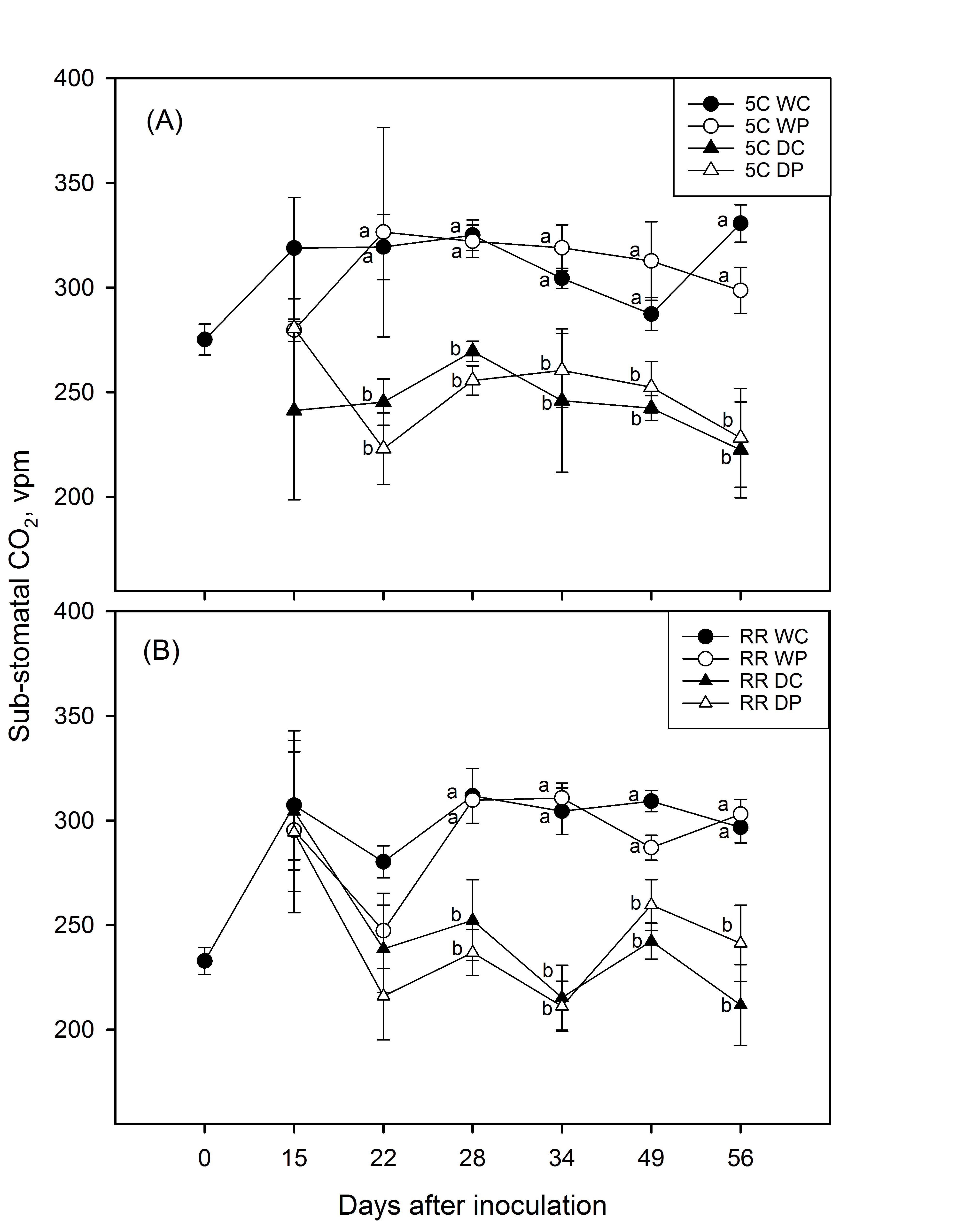


Figure S4

Supplement: Supplementary file 1 — FigureS1. Microclimatic data recorded in the greenhouse during the experimental period: average midday water pressure deficit (grey area, right axis), minimum (closed circles) and maximum (open circles) daily temperatures (left axis). The two arrows indicate the inoculation and the final sampling days, respectively. Figure S2. Stomatal conductance to water vapor (gs) measured in 5C (a) and Riesling grafted on 5C (b) during treatments application (n = 4–7). W = well-watered plants; D = drought-stressed; C = control, non-phylloxerated; P = root phylloxerated. Letters and asterisk indicate statistically significant difference within Irrigation (Factor I; W and D) and Infestation (Factor II; C and P), respectively. No statistically significant interaction between factors was observed. Figure S3. Leaf temperature (Tleaf) measured in 5C (a) and Riesling grafted on 5C (b) during treatments application (n = 4–7). W = well-watered plants; D = drought-stressed; C = control, non-phylloxerated; P = root phylloxerated. Letters denote statistically significant differences within Irrigation (Factor I; W and D). No statistically significant differences within factor Infestation or interaction between factors were observed. Figure S4. Sub-stomatal CO2 (Ci) measured in 5C (a) and Riesling grafted on 5C (b) during treatments application (n = 4–7). W = well-watered plants; D = drought-stressed; C = control, non-phylloxerated; P = root phylloxerated. Letters denote statistically significant differences within Irrigation (Factor I; W and D). No statistically significant differences within factor Infestation or interaction between factors were observed. [file 12870_2019_2017_MOESM1_ESM.doc]
